# Significance of the Number and the Location of Metastatic Lymph Nodes in Locally Recurrent or Persistent Cervical Cancer Patients Treated with Salvage Hysterectomy plus Lymphadenectomy

**DOI:** 10.3390/curroncol29070385

**Published:** 2022-07-11

**Authors:** Seiji Mabuchi, Naoko Komura, Michiko Kodama, Michihide Maeda, Yuri Matsumoto, Shoji Kamiura

**Affiliations:** 1 Department of Gynecology, Osaka International Cancer Institute, 3-1-69, Otemae, Osaka 541-8567, Japan; michihide.maeda@oici.jp (M.M.); kamiura-sh@oici.jp (S.K.); 2 Department of Obstetrics and Gynecology, Kaizuka City Hospital, Kaizuka 597-0015, Japan; naonaokomura@gmail.com; 3 Department of Obstetrics and Gynecology, Osaka University Graduate School of Medicine, Suita 565-0871, Japan; mkodama@gyne.med.osaka-u.ac.jp (M.K.); yuriri49@gmail.com (Y.M.); 4 Department of Obstetrics and Gynecology, Suita Tokusyukai Hospital, Suita 565-0814, Japan

**Keywords:** recurrent cervical cancer, lymph node metastasis, salvage hysterectomy, lymphadenectomy, survival

## Abstract

We retrospectively investigated the significance of metastatic lymph nodes in patients with locally recurrent or persistent cervical cancer in a previously irradiated field and subsequently had salvage hysterectomy. Clinical data were obtained from a chart review, and the prognostic impact of the presence, number (1–2 versus ≥3), and location (pelvic versus pelvic plus para-aortic) of lymph node metastasis was investigated by comparing recurrence and survival. In total, 50 patients were included in this study, of which 21 (42.0%) showed pathological evidence of lymph node metastasis (node-positive group). Both the univariate and multivariate analyses showed that lymph node metastasis was an independent prognostic factor for postoperative recurrence (hazard ratio (HR) 5.36; 95% CI 1.41–6.66; *p* = 0.0020). The predominant sites of recurrence after salvage surgery were the visceral organs and lymph nodes in the node-negative and node-positive groups, respectively. Patients with ≥3 node metastases showed similar survival to those with 1–2 node metastases. Patients with pelvic node metastasis showed similar survival to those with pelvic and para-aortic node metastases. The presence, not number or location, of lymph node metastasis was an independent poor prognostic factor for post-operative recurrence in patients who developed locally recurrent or persistent cervical cancer treated with salvage hysterectomy plus lymphadenectomy.

## 1. Introduction

Approximately one third of patients with invasive cervical cancer develop recurrent disease after primary treatment, usually within 3 years [1]. Previous investigations have suggested that recurrence is localized to the pelvis in approximately 40% of patients previously treated with definitive radiotherapy [2].

Recurrent or persistent cervical cancer in a previously irradiated field is an extremely complicated challenge for gynecological oncologists to face. Although platinum-based combination chemotherapy remains a mainstay in this patient population due to the chemoresistant nature of previously irradiated tumors, patients treated with chemotherapy have a dismal prognosis, with a reported 2-year overall survival rate of approximately 20% [3].

Surgical salvage may be a curative treatment option for this patient population [4,5,6,7,8,9,10,11]. According to previous reports, pelvic exenteration (PE) or radical hysterectomy (RH) results in long-term survival rates of 30–60% [4,5,6,7,8,9,10,11]; however, the post-surgical severe complication rate is 30–60% [4,5,6,7,8,9], while the post-PE mortality rate is 0–17% [10,11]. Considering the highly invasive nature and significant complication rate, identifying a group of patients who would benefit from salvage surgery is important. Recent investigations suggested that incomplete resection, close surgical margins, and parametrial invasion are indicators of poor prognosis in patients with locally recurrent or persistent cervical cancer who had salvage hysterectomy [12].

In newly diagnosed cervical cancer, pelvic node metastasis is an independent predictor of recurrence [13,14,15,16,17]. Moreover, it has been reported that patients with ≥3 pelvic node metastases have higher risk of recurrence than those with 1–2 pelvic node metastasis [13,14,15,16,17]. However, due to the rarity of the cases treated with salvage hysterectomy plus lymphadenectomy, the prognostic significance of the presence, number, or location of lymph node metastases has not been fully investigated in patients who developed locally recurrent or persistent cervical cancer after pelvic radiotherapy.

In this study, we retrospectively evaluated the significance of metastatic lymph nodes in patients who developed locally recurrent or persistent cervical cancer in a previously irradiated field and subsequently had salvage hysterectomy plus lymphadenectomy.

## 2. Materials and Methods

### 2.1. Patients

Permission for data acquisition and analysis was obtained from the Osaka International Cancer Institute Institutional Review Boards. Patients who developed locally recurrent or persistent cervical cancer after definitive radiotherapy and were subsequently treated at our hospitals with salvage hysterectomy, including lymphadenectomy (pelvic and/or para-aortic), between January 2008 and June 2020 were identified and retrospectively reviewed. Patients with visceral metastases, pelvic sidewall recurrence concurrent with cervical tumors, unfavorable histology (small cell carcinoma, glassy cell carcinoma, and undifferentiated carcinoma), or those treated with salvage hysterectomy alone (without lymphadenectomy) were excluded from this study. All surgeries were performed by the two surgeons (S.M. and S.K).

### 2.2. Initial Diagnosis and Radiotherapy

Using biopsy samples obtained from patients prior to the initial treatment, pathologists performed a histological classification of cervical cancer based on the criteria outlined by the World Health Organization (WHO) for uterine cervical tumors [18]. The patients were treated with external beam radiotherapy (EBRT) and high-dose-rate intracavitary brachytherapy (HDR-ICBT) concurrently with platinum-based chemotherapy. EBRT included both whole pelvic and extended-field radiotherapy (EFRT). The planned total doses of EBRT and HDR-ICBT were 50 Gy (in 25 fractions) and 27.2 Gy (in four fractions), respectively. However, in patients who experienced serious radiation-induced toxicities, the radiotherapy dose was reduced as follows: 30 Gy of EBRT in one patient, 40 Gy of EBRT in one patient, and 6.8 Gy of HDR-ICBT in one patient. During and after treatment, patients were regularly followed-up by gynecological oncologists and radiation oncologists, as previously reported [19,20]. When recurrence was suspected, whenever possible, a biopsy was performed for confirmation. Locally recurrent disease was defined as local tumor regrowth identified 3 months or more after the definitive radiotherapy, whereas persistent disease was defined as residual disease identified within 3 months after the definitive radiotherapy.

### 2.3. Salvage Surgery

The pretreatment workup before salvage surgery comprised a complete medical history, a physical examination, a complete blood count, biochemistry panels, chest X-rays, abdominal and pelvic computed tomography (CT) or F-fuoro2-deoxy-d-glucose (FDG) positron-emission tomography and CT (FDG-PET/CT), pelvic magnetic resonance imaging (MRI) including diffusion-weighted imaging, and optional cystoscopy and rectosigmoidoscopy.

Patients with centrally recurrent or persistent cervical cancer underwent salvage surgery via an open approach. Salvage surgeries included either Querlow and Morrow type A–D hysterectomies or pelvic exenteration (PE) with lymphadenectomy (pelvic or pelvic plus para-aortic) [21].

Postoperative adjuvant chemotherapy has been proposed as a treatment option in all cases [7,10]. A total of 21 patients who desired to receive adjuvant chemotherapy were administered paclitaxel (175 mg/m^2^) and, at the physician’s discretion, carboplatin (AUC 5.0, Calvert’s formula) or cisplatin (50 mg/m^2^) within 4–6 weeks after surgery (every 3–4 weeks for a total of three courses).

### 2.4. Assessment of Surgical Complications

Surgical complications were classified according to the Clavien–Dindo system, in which complications are graded from I to V based on the severity and required interventions [22]. Early postoperative and long-term complications were defined as any adverse event that occurred within the first 30 postoperative days and after postoperative day 30, respectively. Treatment-related mortality was defined as any death that occurred within 30 days of surgery and was directly attributable to the surgery itself or any complications.

### 2.5. Follow-Up

Patients were encouraged to undergo regular follow-up in the outpatient unit by gynecological oncologists during and after treatment, as in previous reports [20,23]. Recurrent diseases that developed after salvage surgery were treated according to the institutional guidelines.

### 2.6. Statistical Analysis

Progression-free survival (PFS) was defined as the time from the date of diagnosis of recurrence to the date of the first physical or radiographic evidence of disease progression. Overall survival (OS) was defined as the time from the diagnosis of recurrence to the date of death or last follow-up visit. Continuous data were compared between groups using Student’s *t*-test, the Wilcoxon rank-sum test, or the median test, as applicable. Frequency counts and proportions were compared between groups using chi-square or Fisher’s two-tailed exact tests, as applicable. The survival analysis was based on the Kaplan–Meier method, and results were compared using log-rank tests. All analyses were conducted using JMP version 14.0 (SAS Institute, Cary, NC, USA); *p* < 0.05 was considered statistically significant.

## 3. Results

### 3.1. Prognostic Significance of Nodal Metastasis

Fifty patients with locally recurrent or persistent cervical cancer who had hysterectomy and lymphadenectomy were included in the current study (Table 1). The median age was 53.5 years, and all patients had recurrent (*n* = 13) or persistent (*n* = 37) cervical cancer. Of the 50 patients, 2 received pelvic EBRT alone, and the remaining 48 received pelvic EBRT or EFRT with HDR-ICBT. The original International Federation of Gynecology and Obstetrics (FIGO) stage of the disease was IB−IIA in 5 cases, IIB–IIIA in 22 cases, IIIB-IVA in 17 cases, and IVB in six cases. The median cervical tumor size was 30 mm. Three patients received neoadjuvant chemotherapy. Querlow and Morrow type A hysterectomy was performed in three patients with very small tumors, and a more radical surgery was performed in 47 cases. Pelvic lymphadenectomy (PLND) was performed in 24 patients, and PLN plus para-aortic lymphadenectomy (PALND) was performed in 26 patients. After a median follow-up period of 35 months, 26 patients (52%) developed disease recurrence (Table 2). The median PFS was 34 months. Twenty-four patients (48%) died of their disease, and the median OS was 46 months.

The presence of lymph node metastasis was pathologically demonstrated in 21 patients (node-positive group), including 15 and 6 patients with pelvic lymph node (PLN) metastasis and PLN + para-aortic lymph node (PALN) metastasis, respectively. Comparing clinicopathological characteristics between the node-positive and node-negative groups revealed no significant differences, except for surgical margins, LSVI, or adjuvant treatment after surgery (Supplemental Table S1). A comparison of surgical time, blood loss, blood transfusion, and surgical complications between the node-positive and node-negative groups also revealed no significant differences (Supplemental Table S2).

**Figure 1:**
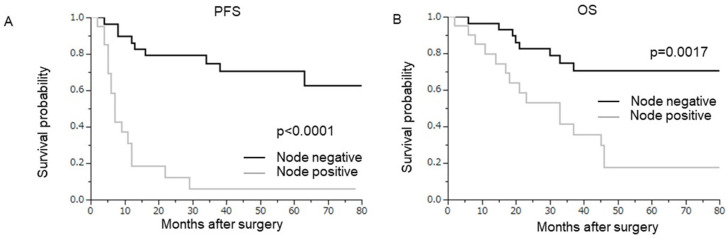
Prognostic significance of lymph node metastasis. Kaplan–Meier estimates of progression-free survival or overall survival according to lymph node status (node-negative (*n* = 29) vs. node-positive (*n* = 21)). (**A**) Progression-free survival (node-negative vs. node-positive; *p* < 0.0001). (**B**) Overall survival (node-negative vs. node-positive; *p* = 0.0017).

In the univariate analysis, in addition to age, tumor diameter, surgical margins, stromal invasion, and perineal cytology, the presence of lymph node metastasis was associated with significantly shorter survival (Table 3, Figure 1A, PFS, *p* < 0.0001; Figure 1B, OS, *p* = 0.0017). In the multivariate analysis, in addition to tumor diameter and surgical margins, lymph node metastasis was found to be an independent poor prognostic factor for post-operative recurrence. The prognostic impact of lymph node metastasis was the greatest among the three independent prognosticators found in the multivariate analysis (Table 3, HR 5.36; 95% CI 1.41–6.66; *p* = 0.0020).

### 3.2. Prognostic Significance of the Number of Lymph Node Metastases

**Figure 2:**
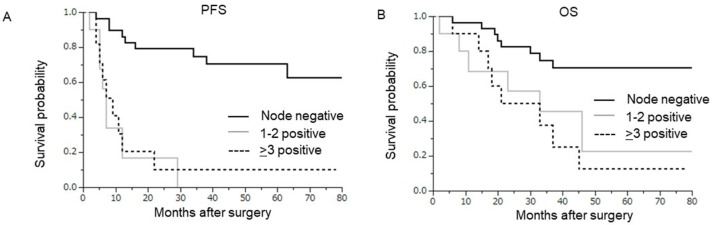
Prognostic significance of the number of lymph node metastases in node-positive patients. Kaplan–Meier estimates of survival according to the number of lymph nodes (1–2 (*n* = 10) versus ≥3 (*n* = 11)). Survival rates of the node-negative group (*n* = 29) are also provided as references. (**A**) Progression-free survival (1–2 versus ≥3, *p* = 0.7736). (**B**) Overall survival (1–2 versus ≥3, *p* = 0.5229).

Of the 21 patients with lymph node metastasis, 10 had 1–2 positive nodes and 11 had ≥3 positive nodes (Table 1). A comparison of the clinicopathological characteristics between the two groups (1–2 vs. ≥3 node metastases) revealed no significant differences in patient characteristics, except for tumor histology, parametrial invasion, surgical margins, and stromal invasion (Supplemental Table S3). As shown in Figure 2, the survival of patients with ≥3 node metastases (≥3) was equivalent to that of patients with 1–2 node metastases (PFS, *p* = 0.7736; OS, *p* = 0.5229).

### 3.3. Prognostic Significance of the Location of Lymph Node Metastases

**Figure 3:**
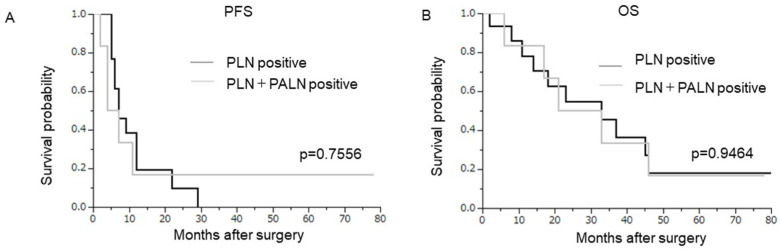
Prognostic significance of the location of lymph node metastases in node-positive patients. Kaplan–Meier estimates of survival according to the location of lymph nodes (PLN (*n* = 15) versus PLN+PALN (*n* = 6)). (**A**) Progression-free survival (PLN versus PLN+PALN, *p* = 0.7556). (**B**) Overall survival (PLN versus PLN+PALN, *p* = 0.9464).

Of the 21 patients with lymph node metastasis, 15 had metastasis in the PLN and 6 had PLN+PALN metastasis (Table 1). Isolated PALN metastasis was not observed. A comparison of the clinicopathological characteristics between the two groups (PLN vs. PLN+PALN) revealed no significant differences in patient characteristics (Supplemental Table S4). As shown in Figure 3, the survival of patients with PLN metastases was equivalent to that of patients with PLN+PALN metastases (PFS, *p* = 0.7556; OS, *p* = 0.9464).

### 3.4. Site of Recurrence after Salvage Surgery According to Lymph Node Metastasis

A total of 9 patients (31%) in the node-negative group and 17 (81%) in the node-positive group developed recurrent disease after salvage surgery, predominantly at distant sites (Table 2). Among patients in the node-negative group, lymph node recurrences were not observed. The predominant sites of recurrence after salvage surgery were the visceral organs and lymph nodes in the node-negative and node-positive groups, respectively.

## 4. Discussion

In this study, we found that the presence and not the number of nodal metastases is an independent predictor of shorter survival in patients with locally recurrent or persistent cervical cancer treated with salvage hysterectomy.

The results of this investigation are partially consistent with those of previous studies on newly diagnosed cervical cancer as follows: the presence of pelvic node metastasis was an independent predictor of shorter survival [13] and was associated with a 20–30% decrease in the 5-year survival rate [13,14,15,16,17]. In this study, as shown in Figure 2, patient survival was unaffected by the number of pelvic lymph node metastases, and patients with 1–2 lymph node metastases had a prognosis similar to that of patients with ≥3 lymph node metastases. This is a clear contrast to patients with newly diagnosed cervical cancer, in whom the number of positive pelvic nodes was shown to be a predictor of shorter survival, and the presence of ≥3 pelvic node metastasis has been consistently associated with an extremely poor prognosis [15,16,17]. This result has several important clinical implications. First, when estimating survival after completing salvage surgery for locally recurrent or persistent cervical cancer, there may be no need to consider the number of positive nodes in this patient population; however, attention should be paid to the presence of nodal metastasis. Moreover, as lymph node recurrence was observed only in node-positive patients, a routine systemic lymph node evaluation may be unnecessary in patients without lymph node metastasis during the follow-up period after salvage hysterectomy.

In our patients who underwent salvage hysterectomy plus lymphadenectomy, the rate of grade 3–4 complications was 28% (Supplemental Table S2). Considering the high incidence of postoperative complications, lymphadenectomy should be performed sparingly or should be performed in the context of a clinical trial of sentinel lymph node (SLN) mapping in patients who undergo salvage hysterectomy. Moreover, given the high probability of treatment failure, surgical salvage should be offered, ideally, for a select group of patients with recurrent or persistent cervical cancer who have a high probability of benefitting from salvage surgery—patients without lymph node metastasis. In this study, all patients underwent preoperative imaging studies (pelvic MRI plus the physician’s choice of either abdominal CT or FDG-PET/CT). However, among the 24 patients who had preoperative radiological evidence of lymph node metastasis, lymph node metastasis could not be pathologically demonstrated in 9 (37.5%) cases (data not shown). Meanwhile, among the 26 patients without preoperative radiological evidence of lymph node metastasis, lymph node metastasis was pathologically demonstrated in 5 (19.2%) cases (data not shown). These results are in line with previous studies that indicated the difficulty in diagnosing the presence of lymph node metastasis in this patient population and selecting the best candidates for salvage hysterectomy (i.e., patients without lymph node metastasis) using the current imaging techniques [24]. Thus, it is important to develop more reliable methods of detecting lymph node metastasis, i.e., new imaging techniques or SLN mapping.

To further improve the oncological outcomes of salvage hysterectomy plus lymphadenectomy in node-positive patients, we examined the pattern of recurrence and found distant areas to be the predominant site of recurrence in both node-negative and node-positive patients (Table 3). Thus, adjuvant chemotherapy after salvage surgery may be a reasonable treatment strategy for prolonging survival. In the present study, 14 of 21 patients in the node-positive group received adjuvant chemotherapy. However, due to the lack of clinical evidence supporting the benefit of adjuvant chemotherapy in the setting of salvage hysterectomy, the remaining seven patients did not agree to receive adjuvant chemotherapy (Supplemental Table S3). The employed regimen was paclitaxel and, at the investigator’s discretion, cisplatin or carboplatin. Since approximately 15% of Japanese patients with recurrent cervical cancer and a history of pelvic radiotherapy developed gastrointestinal perforation or fistula after receiving bevacizumab-containing chemotherapy [25], we did not use bevacizumab-containing regimens as adjuvant chemotherapy after salvage surgery. However, as pembrolizumab’s efficacy was recently demonstrated in patients with recurrent or advanced cervical cancer [26], the efficacy of adjuvant chemotherapy comprising platinum-based chemotherapy with or without bevacizumab or pembrolizumab should be evaluated in the future.

Our study had several limitations. First, the sample size was relatively small. Next was its retrospective design and potential for selection bias from physicians selecting surgery as a salvage treatment. Third, the study population was heterogeneous—the investigation included both patients with recurrence and those with persistent cervical cancer who may have had different clinical characteristics. Moreover, the disease-free interval (the time from radiotherapy to the diagnosis of local recurrence) may also affect the posttreatment survival. Thus, the significance of lymph node metastasis should be further evaluated in a prospective study with a larger number of patients. Finally, we have to recognize that the patient population and the type of salvage surgery that was selected for this study may not be applicable to the general practice: patients highly suspected for nodal metastasis were usually excluded as candidates for salvage hysterectomy, and PE instead of radical hysterectomy is the most popular procedure for locally recurrent or persistent cervical cancer.

In conclusion, we found that the presence and not the number of lymph node metastases was an independent indicator of post-operative recurrence and shorter survival in patients who developed locally recurrent or persistent cervical cancer in a previously irradiated field and subsequently had salvage hysterectomy.

## Figures and Tables

**Table 1:** Clinicopathological characteristics of the patients.

		All Patients (*n* = 50) *n* (%)
Initial characteristics		
Initial stage ^a^	IB1-IIA2	5 (10.0)
	IIB-IIIA	22 (44.0)
	IIIB-IVA	17 (34.0)
	IVB	6 (12.0)
Histology	SCC	30 (60.0)
	AC	20 (40.0)
Types of radiotherapy	Pelvic EBRT+ICBT	44 (88.0)
	EFRT+ICBT	4 (8.0)
	Pelvic ERBT alone	2 (4.0)
Concurrent chemotherapy	Yes	48 (96.0)
	No	2 (4.0)
Post-recurrence characteristics		
Age	Median (range)	53.5 (26–82)
	≤39	8 (16.0)
	40–64	35 (70.0)
	65≤	7 (14.0)
Disease status	Recurrent cancer	13 (26.0)
	Persistent cancer	37 (74.0)
BMI	Median (range)	20.4 (13.6–32.2)
	<20.0	23 (46.0)
	≥20.0	27 (54.0)
Tumor diameter (mm) ^b^	Median (range)	30 (5–75)
	<10	12 (24.0)
	10–20	4 (8.0)
	20≤	34 (68.0)
NAC	No	47 (94.0)
	Yes	3 (6.0)
Hysterectomy performed	Type A	3 (6.0)
	Type B or greater	47 (94.0)
Lymphadenectomy performed	PLND	24 (48.0)
	PLND+PALND	26 (52.0)
Pathological findings from salvage surgery		
Parametrial invasion	Negative	35 (70.0)
	Positive	15 (30.0)
Surgical margins	Negative	38 (76.0)
	Close or positive	12 (24.0)
Location of lymph node metastasis	No	29 (58.0)
	PLN	15 (30.0)
	PLN+PAN	6 (12.0)
Number of lymph node metastases	0	29 (58.0)
	1–2	10 (20.0)
	3≤	11 (22.0)
Stromal invasion	Less than half	22 (44.0)
	More than half	28 (56.0)
LSVI	Negative	25 (50.0)
	Positive	25 (50.0)
Peritoneal cytology ^c^	Negative	44 (88.0)
	Positive	2 (4.0)
Adjuvant treatments after surgery	No	29 (58.0)
	Yes	21 (42.0)
Symptom status	No	29 (58.0)
	Yes	21 (42.0)

SCC, squamous cell carcinoma; AC, adenocarcinoma; EBRT, external beam radiotherapy; ICBT, intracavitary brachytherapy; EFRT, extended-field radiotherapy; NAC, neoadjuvant chemotherapy; PLN, pelvic lymph nodes; PLND, pelvic lymphadenectomy; PALND, para-aortic lymphadenectomy. ^a^ FIGO 2008 staging system. ^b^ Assessed using preoperative pelvic MRI at the time of recurrence diagnosis. ^c^ Four patients who did not undergo peritoneal cytology were excluded.

**Table 2:** Treatment outcomes.

			All Patients (*n* = 50) *n* (%)	Node-Negative Group (*n* = 29) *n* (%)	Node-Positive Group (*n* = 21) *n* (%)	*p*-Value
Recurrence after surgery	No		24 (48.0)	20 (69.0)	4 (19.1)	0.0005
	Yes (median 34 months)		26 (52.0)	9 (31.0)	17 (81.0)	
Site of recurrence after surgery	Pelvis alone		5 (19.2)	1 (11.1)	4 (23.5)	0.4447
	Distant alone or Pelvis+Distant	All	21 (80.8)	8 (88.9)	13 (76.5)	
		LNs ^a^	6 (23.1)	0	6 (35.3)	0.0737
		Disseminations ^b^	6 (23.1)	3 (33.3)	3 (17.6)	
		Visceral organs ^c^	9 (34.6)	5 (55.6)	4 (23.5)	
Deaths after surgery	No		26 (52.0)	20 (69.0)	6 (28.6)	0.0048
	Yes (median 46 months)		24 (48.0)	9 (31.0)	15 (71.4)	

LN; lymph node. ^a^ Lymph node recurrence with or without visceral metastasis. ^b^ Disseminations with or without visceral metastasis. ^c^ Visceral metastases alone.

**Table 3:** Univariate and multivariate analysis of prognostic factors for post-operative recurrence.

		Univariate Analysis			Multivariate Analysis		
		Hazard Ratio	95%CI	*p*-Value	Hazard Ratio	95%CI	*p*-Value
Age (years)	<50	1			1		
	50≤	5.24	1.45–23.30	0.0098	1.81	0.74–4.80	0.1996
BMI	<20.0	1					
	≥20.0	0.67	0.21–2.10	0.4839			
Initial stage ^a^	IB1-IIA	1					
	IIB-IIIA	0.45	0.07–3.92	0.4276			
	IIIB-IVA	0.51	0.06–7.51	0.5947			
	IVB	8.82	0.74–175.2	0.0865			
Histology	SCC	1					
	Non-SCC	1.64	0.30–8.87	0.5555			
Disease status	Recurrent	1					
	Persistent	5.32	0.83–31.45	0.0756			
Tumor diameter ^b^	<2 cm	1			1		
	≥2 cm	13.91	2.06–135.4	0.0058	5.11	1.45–24.32	0.0100
NAC	No	1					
	Yes	0.28	0.02–3.21	0.2997			
Hysterectomy performed	Type A	1					
	Type B or greater	12.86	0.23–1314.91	0.2221			
Lymphadenectomy performed	PLND	1					
	PLND+PALND	1.41	0.65–3.23	0.3884			
Parametrial invasion	Negative	1					
	Positive	0.35	0.04–2.36	0.2805			
Surgical margins	Negative	1			1		
	Close or positive	11.41	1.32–121.03	0.0268	4.49	1.27–16.94	0.0197
Lymph node metastasis	Negative	1			1		
	Positive	7.57	3.27–18.67	<0.0001	5.36	1.41–6.66	0.0020
Stromal invasion	Less than half	1			1		
	More than half	6.89	1.02–50.42	0.0472	2.44	0.79–8.51	0.1240
LSVI	Negative	1					
	Positive	1.45	0.13–13.98	0.7556			
Peritoneal cytology ^c^	Negative	1			1		
	Positive	51.9	4.17–1277.18	0.0014	1.79	0.39–12.99	0.4759
Adjuvant treatments after hysterectomy	No	1					
	Yes	1.75	0.27–10.55	0.5460			
Symptom status	No	1					
	Yes	1.32	0.31–5.80	0.6966			

SCC, squamous cell carcinoma; NAC, neoadjuvant chemotherapy; BMI, body mass index; PLND, pelvic lymphadenectomy; PALND, para-aortic lymphadenectomy. ^a^ FIGO 2008 staging system. ^b^ Assessed using preoperative pelvic MRI at the time of recurrence diagnosis. ^c^ Four patients who did not undergo peritoneal cytology were excluded.

## Data Availability

Not applicable.

## Supplementary Materials

The following supporting information can be downloaded at: https://www.mdpi.com/article/10.3390/curroncol29070385/s1, Table S1: Clinicopathological characteristics of patients according to lymph node metastasis, Table S2: Surgical outcomes, Table S3: Clinicopathological characteristics of patients according to the number of lymph node metastases, Table S4: Clinicopathological characteristics of patients according to the location of lymph node metastases.
