# Supplementary material for: Significance of the Number and the Location of Metastatic Lymph Nodes in Locally Recurrent or Persistent Cervical Cancer Patients Treated with Salvage Hysterectomy plus Lymphadenectomy
Source: Curr Oncol. 2022 Jul 11;29(7):4856–67. doi: 10.3390/curroncol29070385 (PMC9315867; doi:10.3390/curroncol29070385)
Supplement: Supplementary file 1 [file curroncol-29-00385-s001.zip › curroncol-1755572-SI.pdf]

Table S1. Clinicopathological characteristics of the patients according to lymph node metastasis.

		Node-negative group (n=29) N (%)	Node-positive group (n=21) N (%)	P-value
Initial characteristics				
Initial stage [a]	IB1-IIA2	4 (13.8)	1 (4.8)	0.3982
	IIB-IIIA	14 (48.3)	8 (38.1)	
	IIIB-IVA	9 (31.0)	8 (38.1)	
	IVB	2 (6.9)	4 (19.1)	
Histology	SCC	15 (51.7)	15 (71.4)	0.1604
	AC	14 (48.3)	6 (28.6)	
Types of radiotherapy	Pelvic EBRT + ICBT	25 (86.2)	19 (90.5)	0.4542
	EFRT + ICBT	2 (6.9)	2 (9.5)	
	Pelvic ERBT alone	2 (6.9)	0	
Concurrent chemotherapy	Yes	28 (96.6)	20 (95.2)	0.8150
	No	1 (3.5)	1 (4.8)	
Post recurrence characteristics				
Age	≤ 39	5 (17.2)	3 (14.3)	0.9612
	40-64	20 (69.0)	15 (71.4)	
	65 ≤	4 (13.8)	3 (14.3)	
Disease status	Recurrent cancer	8 (27.6)	5 (23.8)	0.7638
	Persistent cancer	21 (72.4)	16 (76.2)	
BMI	< 20.0	12 (41.4)	11 (52.4)	0.4411
	≥ 20.0	17 (58.6)	10 (47.6)	
Tumor diameter (mm) [b]	< 10	10 (34.5)	2 (9.5)	0.0704
	10-20	3 (10.3)	1 (4.8)	
	20 ≤	16 (55.2)	18 (85.7)	
NAC	No	27 (93.1)	20 (95.2)	0.7538
	Yes	2 (6.9)	1 (4.8)	
Surgery performed	Piver type I	1 (3.5)	2 (9.5)	0.3719
	Piver type II or grater	28 (96.6)	19 (90.5)	
Pathological findings from salvage surgery				
Parametrial invasion	Negative	23 (79.3)	12 (57.1)	0.0914
	Positive	6 (20.7)	9 (42.9)	
Surgical margins	Negative	26 (89.6)	12 (57.1)	0.0079
	Close or positive	3 (10.3)	9 (42.9)	
Stromal invasion	Less than half	14 (48.3)	8 (38.1)	0.4741
	More than half	15 (51.7)	13 (61,9)	
LSVI	Negative	18 (62.1)	7 (33.3)	0.0449
	Positive	11 (37.9)	14 (66.7)	
Peritoneal cytology [c]	Negative	27 (100)	17 (89.5)	0.0848
	Positive	0	2 (10.5)	
Adjuvant treatments after surgery	No	22 (75.9)	7 (33.3)	0.0026
	Yes	7 (24.1)	14 (66.7)	
Symptom status	No	17 (58.6)	12 (57.1)	0.9168
	Yes	12 (41.4)	9 (42.9)	

SCC, squamous cell carcinoma; AC, adenocarcinoma; EBRT, external beam radiotherapy; ICBT, intracavitary brachytherapy; EFRT, extended-field radiotherapy; NAC, neoadjuvant chemotherapy; LVSI, lymphovascular space involvement; BMI, body mass index.

[a] FIGO 2008 staging system.

[b] Assessed using preoperative pelvic MRI at the time of recurrence diagnosis.

[c] Four patients who did not undergo peritoneal cytology were excluded.

Table S2. Surgical outcomes

		All patients (N=50) N (%)	Node-negative group (N=29) N (%)	Node-positive group (N=21) N (%)	P-value
Surgical time (min)	Median (range)	460 (155-729)			
	< 480	32 (64.0)	20 (69.0)	12 (57.1)	0.3900
	≥ 480	18 (36.0)	9 (31.0)	9 (42.9)	
Blood loss (ml)	Median (range)	1350 (280-5335)			
	< 1500	27 (54.0)	18 (62.1)	9 (42.9)	0.1785
	≥ 1500	23 (46.0)	11 (37.9)	12 (57.1)	
Blood transfusion	No	26 (52.0)	17 (58.6)	9 (42.9)	0.2708
	Yes	24 (48.0)	12 (41.4)	12 (57.1)	
Surgical complications (Grade 3-4)	Early complications	2 (4.0)	1 (3.5)	1 (4.8)	0.8150
	Late complications	14 (28.0)	9 (31.0)	5 (23.8)	0.5744

Table S3. Clinicopathological characteristics of the patients according to the number of lymph node metastasis.

		1-2 nodes metastasis (n=10) N (%)	Multiple (≥3) nodes metastasis (n=11) N (%)	P-value
Initial characteristics				
Initial stage [a]	IB1-IIA2	1 (10.0)	0	0.5814
	IIB-IIIA	4 (40.0)	4 (36.4)	
	IIIB-IVA	4 (40.0)	4 (36.4)	
	IVB	1 (10.0)	3 (27.3)	
Histology	SCC	10 (100)	5 (45.5)	0.0057
	AC	0	6 (54.6)	
Types of radiotherapy	Pelvic EBRT + ICBT	9 (90.0)	10 (90.9)	0.9435
	EFRT + ICBT	1 (10.0)	1 (9.1)	
	Pelvic ERBT alone	0	0	
Concurrent chemotherapy	Yes	9 (90.0)	11(100)	0.2825
	No	1 (10.0)	0	
Post recurrence characteristics				
Age	≤ 39	2 (20.0)	1 (9.1)	0.7092
	40-64	7 (70.0)	8 (72.7)	
	65 ≤	1 (10.0)	2 (18.2)	
Disease status	Recurrent cancer	6 (60.0)	10 (90.9)	0.0967
	Persistent cancer	4 (40.0)	1 (9.1)	
BMI	< 20.0	6 (60.0)	5 (45.5)	0.5051
	≥ 20.0	4 (40.0)	6 (54.6)	
Tumor diameter (mm) [b]	< 10	2 (20.0)	0	0.2037
	10-20	0	1 (9.1)	
	20 ≤	8 (80.00)	10 (90.9)	
NAC	No	9 (90.0)	11 (100)	0.2825
	Yes	1 (10.0)	0	
Surgery performed	Piver type I	0	2 (18.2)	0.1563
	Piver type II or grater	10 (100)	9 (81.8)	
Pathological findings from salvage surgery				
Parametrial invasion	Negative	8 (80.0)	4 (36.4)	0.0436
	Positive	2 (20.0)	7 (63.6)	
Surgical margins	Negative	8 (80.0)	4 (36.4)	0.0436
	Close or positive	2 (20.0)	7 (63.6)	
Stromal invasion	Less than half	7 (70.0)	1 (9.1)	0.0041
	More than half	3 (30.0)	10 (90.9)	
LSVI	Negative	4 (40.0)	3 (27.3)	0.5366
	Positive	6 (27.3)	8 (72.7)	
Peritoneal cytology [c]	Negative	9 (90.0)	8 (88.9)	0.9372
	Positive	1 (10.0)	1 (11.1)	
Adjuvant treatments after surgery	No	5 (50.0)	2 (18.2)	0.1224
	Yes	5 (50.0)	9 (81.8)	
Symptom status	No	6 (60.0)	6 (54.6)	0.8008
	Yes	4 (40.0)	5 (45.5)	

SCC, squamous cell carcinoma; AC, adenocarcinoma; EBRT, external beam radiotherapy; ICBT, intracavitary brachytherapy; EFRT, extended-field radiotherapy; NAC, neoadjuvant chemotherapy; LVSI, lymphovascular space involvement; BMI, body mass index.

[a] FIGO 2008 staging system.

[b] Assessed using preoperative pelvic MRI at the time of recurrence diagnosis.

[c] Four patients who did not undergo peritoneal cytology were excluded.

Table S4. Clinicopathological characteristics of the patients according to the location of lymph node metastasis.

		PLN alone (n=15) N (%)	PLN+PALN (n=6) N (%)	P-value
Initial characteristics				
Initial stage [a]	IB1-IIA2	1 (6.7)	0	0.1278
	IIB-IIIA	7 (46.7)	1 (16.7)	
	IIIB-IVA	6 (40.0)	2 (33.3)	
	IVB	1 (6.7)	3 (50.0)	
Histology	SCC	12 (80.0)	3 (50.0)	0.1692
	AC	3 (20.0)	3 (50.0)	
Types of radiotherapy	Pelvic EBRT + ICBT	14 (93.3)	5 (83.3)	0.4807
	EFRT + ICBT	1 (6.7)	1 (16.7)	
	Pelvic ERBT alone	0	0	
Concurrent chemotherapy	Yes	14 (93.3)	6 (100)	0.5169
	No	1 (6.7)	0	
Post recurrence characteristics				
Age	≤ 39	2 (13.3)	1 (16.7)	0.4966
	40-64	10 (66.7)	5 (83.3)	
	65 ≤	3 (20.0)	0	
Disease status	Recurrent cancer	4 (26.7)	1 (16.7)	0.6269
	Persistent cancer	11 (73.3)	5 (83.3)	
BMI	< 20.0	8 (53.3)	3 (50.0)	0.8901
	≥ 20.0	7 (46.7)	3 (50.0)	
Tumor diameter (mm) [b]	< 10	2 (13.3)	0	0.1915
	10-20	0	1 (16.7)	
	20 ≤	13 (86.7)	5 (83.3)	
NAC	No	14 (93.3)	6 (100)	0.5169
	Yes	1 (6.7)	0	
Surgery performed	Piver type I	2 (13.3)	0	0.3471
	Piver type II or grater	13 (86.7)	6 (100)	
Pathological findings from salvage surgery				
Parametrial invasion	Negative	9 (60.0)	3 (50.0)	0.6757
	Positive	6 (40.0)	3 (50.0)	
Surgical margins	Negative	10 (66.7)	2 (33.3)	0.1632
	Close or positive	5 (33.3)	4 (66.7)	
Stromal invasion	Less than half	7 (46.7)	1 (16.7)	0.2009
	More than half	8 (53.3)	5 (83.3)	
LSVI	Negative	5 (33.3)	2 (33.3)	1.0000
	Positive	10 (66.7)	4 (66.7)	
Peritoneal cytology [c]	Negative	13 (92.9)	4 (80.0)	0.4213
	Positive	1 (7.1)	1 (20.0)	
Adjuvant treatments after surgery	No	6 (40.0)	1 (16.7)	0.3055
	Yes	9 (60.0)	5 (83.3)	
Symptom status	No	10 (66.7)	2 (33.3)	0.1632
	Yes	5 (33.3)	4 (66.7)	

SCC, squamous cell carcinoma; AC, adenocarcinoma; EBRT, external beam radiotherapy; ICBT, intracavitary brachytherapy; EFRT, extended-field radiotherapy; NAC, neoadjuvant chemotherapy; LVSI, lymphovascular space involvement; BMI, body mass index..

[a] FIGO 2008 staging system.

[b] Assessed using preoperative pelvic MRI at the time of recurrence diagnosis.

[c] Two patients who did not undergo peritoneal cytology were excluded.
